# A contemporary insight into the sero-epidemiology of *Toxoplasma gondii* infection in the foot-hills of Himalayas: A cross-sectional study from a tertiary care center in Northern India

**DOI:** 10.3126/nje.v11i1.34228

**Published:** 2021-03-31

**Authors:** Sangeeta Deka, Deepjyoti Kalita, Pratima Gupta, Yogendra Pratap Mathuria

**Affiliations:** 1 Department of Microbiology, All India Institute of Medical Sciences, Rishikesh, Uttarakhand, India; 2 Fakhruddin Ali Ahmed Medical College and Hospital, Barpeta, Assam, India

**Keywords:** Hilly area, Risk factors, Seroprevalence, Toxoplasmosis, Uttarakhand

## Abstract

**Background:**

*Toxoplasma gondii* infects 30-50% of the world’s population with high diversity in the geo-epidemiology and seroprevalence. The burden of toxoplasmosis and its determinants from remote and vulnerable regions of India is unknown. Therefore, this study aim to evaluate the prevalence of toxoplasma antibodies and factors associated with seropositivity among individuals from Uttarakhand and adjoining areas.

**Methods:**

Serum samples from 442 cases were tested for anti-Toxoplasma IgG and IgM antibodies by Enzyme-linked Immunosorbent Assay. Association of seropositivity of toxoplasmosis with age, gender, place of residence, dietary habits, and comorbidities were analyzed using binary logistic regression analysis.

**Results:**

The overall Toxoplasma seropositivity was found to be 36.88% [95% Confidence Interval (CI)=30.40–39.28]. While anti-T. gondii IgG was present in 34.84% [95% CI=30.40–39.28], anti-IgM was detected in 6.33% [95% CI=4.06–8.61]. The overall and IgG seroprevalence increased with age in both the genders and there was a sharp increase in the seropositivity after the age of 40 years [adjusted Odds Ratio (aOR)=2.98, p-value=0.030]. The seropositivity rate was significantly higher in individuals from Uttarakhand in both the hilly region (aOR=5.61; 95%CI:[2.14-14.68]) and plains (aOR=5.14; 95%CI:[2.2-12.02]). Multivariable logistic regression analysis also showed that being rural residents (aOR=3.23; 95%CI:[1.67-6.23]) and presence of comorbidities (aOR=8.64; 95%CI:[4.62-16.18]) were potential risk factors of Toxoplasmosis. On the other hand, consumption of vegetarian diet was found to have a protective effect (aOR=0.46; 95%CI:[0.28-0.75]).

**Conclusion:**

Seroprevalence of T. gondii antibodies was relatively high in Uttarakhand, particularly in rural and hilly terrain, indicating a necessity for the implementation of integrated public health strategies to prevent and control toxoplasmosis in this region.

## Introduction

Toxoplasma gondii (T. gondii) is an obligate intracellular apicomplexan parasite which is distributed worldwide among all warm-blooded animals and affects nearly one-third of the world population [1]. More than 350 host species, mammals and birds (mostly wild animals) have been identified to harbor T. gondii as an intermediate host and there is evidence of high seroprevalence in wild felids which may reach up to 100% [2].

Globally, Toxoplasma seroprevalence is estimated to be 30-50% of the world population making it the most prevalent infection in humans [3]. However, seroprevalence of the parasite measured by specific anti-T. gondii antibodies reveals wide variability that ranges between 1% to 100% depending upon the climatic conditions, socioeconomic status including personal hygiene, cultural beliefs, dietary habits and other anthropogenic factors [2,3]. The incidence of infection is higher in areas with warmer and humid climates and increases with age. In India, the seroprevalence was found to be as high as 24.3% (IgG antibody). However, depending upon the geographic heterogeneity and biodiversity it varies widely[4,5]. For instance, in Northern India, it ranged from 9.4-19.7%, and in South India, a relatively higher seroprevalence of 21.8-48.2% had been reported [4-7]. Toxoplasma-related mortality amongst Human Immunodeficiency Virus (HIV) positive cases were reported to be 10-30% and 20-50% in the US and Europe, respectively [8]. An earlier study from Tanzania, showed that the age-standardized mortality rate per 100,000 population had increased substantially from 0.11 in 2006 to 0.79 in 2015 [9]. Notably, Toxoplasmosis is an important cause of preventable deaths, especially among non-HIV population.

Toxoplasmosis infection is mainly caused by ingestion of T. gondii oocyst present on raw and unwashed vegetables or undercooked meat containing tissue cysts [10]. Both acute infection and reactivation of past infection are possible, and in either case, clinical spectrum may range from asymptomatic/mild non-specific flu-like symptoms to severe lethal forms like cerebral, pulmonary or disseminated toxoplasmosis depending upon immune status of the patients [11,12]. Besides, there is evidence of correlation between toxoplasma seroprevalence and specific disease burden indicating the possible role of toxoplasmosis as a triggering factor responsible for the development of several clinical entities [3].

The high prevalence of toxoplasmosis with its significant contribution to morbidity and mortality, together with inappropriate diagnosis, management and prevention often results in immense public health impact. Therefore, prevention of Toxoplasmosis is an important aspect to minimize the disease burden. But due to lack of a national screening program in India, there is paucity of data on baseline seropositivity and associated risk factors especially in remote, vulnerable populations. Knowledge of the demographic characteristics and seroprevalence of toxoplasmosis in a given location may help in developing extensive diagnostic facilities, intervention/preventive measures and public health policies. The present study was undertaken to detect the seroprevalence of T. gondii among inhabitants of Uttarakhand and adjoining areas, and to identify its association with socio-demographic characteristics, epidemiological profile and possible risk factors of toxoplasmosis.

## Methodology

### Study design and participants

This is a hospital-based, cross-sectional study conducted at All India Institute of Medical Sciences, Rishikesh from March 2015 to December 2019. This hospital renders healthcare services to the people residing in the plains and hilly terrain of Uttarakhand and the neighboring states mainly Western Uttar Pradesh, Himachal Pradesh, Haryana and Rajasthan.

### Data Collection

Blood specimens were drawn under aseptic conditions by venipuncture which was collected in sterile plain vials. Serum samples were separated through centrifugation which was later aliquoted and stored at -200C until further investigation. Serum samples were tested for the presence of specific anti-T. gondii IgG antibodies using a commercial quantitative enzyme-linked immunosorbent assay (Calbiotech Toxoplasma IgG R5EC 96 well ELISA Kit, California, USA). To detect newly acquired infection the IgM antibodies capture ELISA (Calbiotech Toxoplasma IgM R5EC 96 well ELISA, California, USA) was performed with a sensitivity and specificity of 95.8% and 89.7% respectively and 98% agreement with a reference ELISA method. All samples and ELISA kit reagents were brought to ambient temperature and the ELISA test was performed using automated Euroimmune Analyzer 1 (Walkaway automated seven plate ELISA reader, Euroimmune, Germany). Socio-demographic details such as age, gender and place of residence of the patients who had undergone testing for the anti-toxoplasmosis antibodies (IgM & IgG) were extracted from the medical records maintained in the department.

### Inclusion and exclusion criteria

All samples received in the Serology section of Microbiology department with clinical suspicion of toxoplasmosis and those undergoing routine screening (antenatal) were included in the study. Unlabeled, inadequate, hemolyzed, lipemic, and leaking samples were excluded. Also, cases with missing data were excluded. Antibody index of >1.1 was interpreted as detectible Toxoplasma IgM and IgG. Antibody index of 0.9-1.1 were regarded as equivocal for both IgM and IgG and were excluded from the study. Clinical information such as present illness, history of past illness, dietary habits and findings on general examination were also obtained from the patient’s medical history sheets.

### Outcome Variable

Seropositivity for anti-T. gondii IgM and IgG antibodies based on ELISA

### Explanatory Variable

Socio-demographic variables (such as age, gender, religion), place of residence (state, rural/urban), dietary habits and clinical variable (presence of co-morbidities).

### Ethical Clearance

The study was approved by the Institutional Ethics Committee of our institute (Letter No-AIIMS/IEC/IEC/20/835 Date: 12/12/2020). In order to maintain confidentiality, name and registration numbers were not included while compiling the data, and instead each case was given a unique identifier number.

### Sample size calculation

A nationwide survey on serological prevalence of Toxoplasma gondii in India reported a rate of 24.3% [4]. Using the formula, n = z^2^ *P(1-P)/d^2^ [n = sample size; z =confidence level at 95% (standard value of 1.96); P = expected prevalence of the disease in the particular area; d = precision] and taking taking 4% as absolute precision, the sample size was calculated to be 442.

### Data management and statistical analysis

Data were entered from medical records into Microsoft Office Excel spreadsheet 2010 by two researchers individually and were verified for accuracy by a third researcher (compared with the pre-existing data in the registers and patient history sheets). Statistical analyses were carried out using IBM SPSS Statistics version 23.0 (Armonk, NY: IBM Corp.). Period prevalence estimates with 95% Confidence Intervals (CI) were calculated for overall, IgG and IgM seropositivity and in each individual group. The dependent variable was converted to dichotomous outcome (Positive or negative for Toxoplasma IgG/IgM antibodies). Based on available data, the independent variables such as age (years), different age groups, gender, religion, place of residence, rural/urban, dietary habits and co-morbidity were selected for testing their putative association with toxoplasma seropositivity. We have investigated the association between explanatory variables and seropositivity by performing stepwise conditional logistic regression analysis. All risk factors with a P-value <0.1 at the bivariate level were included in the multivariate logistic model. Odds ratios (OR) and respective 95% confidence intervals (CI95%) were estimated for each level. P-value <0.05 was regarded as statistically significant at each level.

## Results

### Study participation and population characteristics

**Figure 1:**
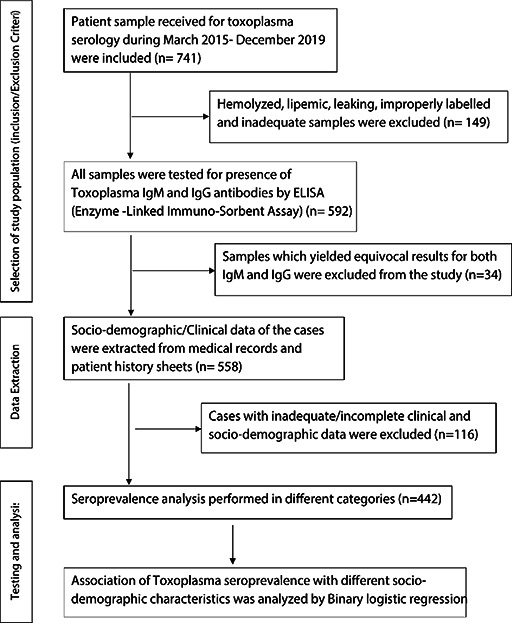
Flow diagram of the study design depicting the enrolment of cases

A total of 741 cases were initially included in the study, of which 299 were excluded due to various reasons (Figure 1). Of the finally enrolled 442 cases, 220 (49.77%) were male and 222 (50.23%) were females. Mean age of the study population was 18.65 + 2.42 years; with age ranged between 4 days after birth to 71 years. 57.24% (n=253) cases were from Uttarakhand (Hills: n=114; Plains: n=139), 29.19% (n=129) were from the Western Uttar Pradesh (UP) and 13.57% (n=60) were from the adjacent states including Himachal Pradesh, Rajasthan, Haryana and Punjab. 66.52% of cases resided in rural areas while 33.48% hailed from Urban places. Majority practiced Hinduism (82.8%) while 14.5% were Muslims and 2.7% practiced other religions (Sikh, Christianity, and Buddhism). Single or multiple co-morbidities like Diabetes, HIV infection, past history of tuberculosis, SLE, viral hepatitis, congenital heart defects, other congenital anomalies, neoplasia and thalassemia were identified in 19% cases.

### Seropositivity of anti-Toxoplasma antibodies

**Figure 2:**
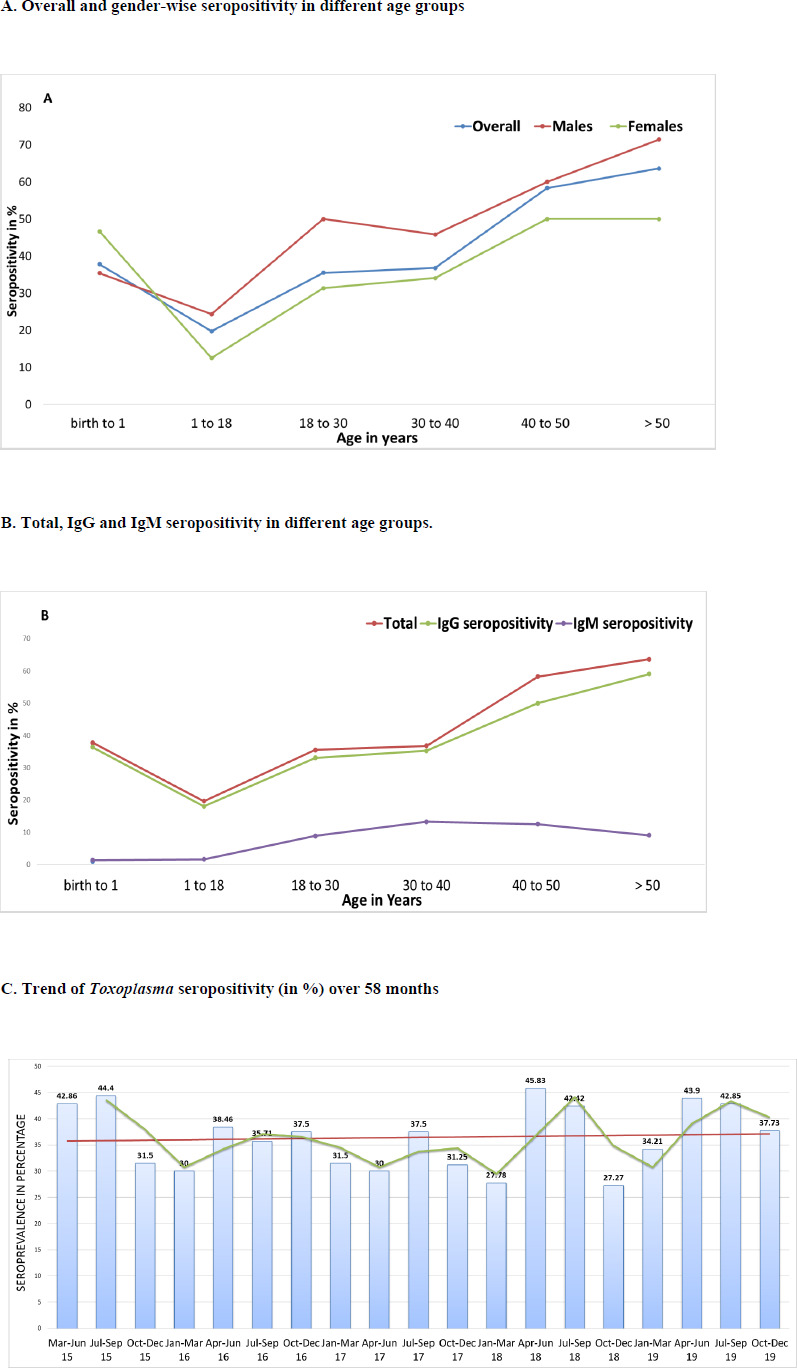
Percentage prevalence of *Toxoplasma gondii* seropositivity:

Table 1 shows the seroprevalence of IgG and IgM antibodies to Toxoplasma gondii among the study population. The overall T. gondii seroprevalence was 36.88%, with 34.84% being positive for anti-T. gondii IgG and 6.33% seropositive for anti-T. gondii IgM only and 4.3% positive for both IgG and IgM (Table 1). Figure 2A-C demonstrates the percentage prevalence of Toxoplasma gondii seropositivity. Figure 2A shows the percentage of seropositivity in males and females among different age groups. Higher seropositivity was observed in female infants as compared to males (46.7% vs 35.4%). From 1 year onwards a linear increase in seropositivity was observed in both the genders and the trend increases with increasing age. A similar trend was also observed for both IgG and IgM seropositivity individually (Figure 2B). The trend of percentage seropositivity of T. gondii over 58 months is depicted in Figure 2C. While the overall seropositivity remained steady over the 5 years, a dropto 27.27 to 31.50% was observed in the colder months of October to March as compared to summer season (Figure 2C).

### Toxoplasma seropositivity and associated factors

Table 2 shows the stratified seroprevalence of anti-T. gondii antibodies and results of binomial logistic regression analysis of potential risk factors for seropositivity. A significant association of Toxoplasma seropositivity was noticed with increasing age (p-value:0.012). Higher positivity of 37.8% was found among infants (<1year) and keeping it as reference it was observed that seropositivity was significantly less in the pediatric age group (1-18 years) (p-value:0.013). In adults, seropositivity increased gradually after 18 years and there was 2.31- and 2.88-times higher odds of having seropositivity in the age group 40-50 years and >50 years respectively.

Taking seropositivity of cases from adjacent states as reference, there was strikingly high seropositivity in the state of Uttarakhand, especially in the hilly terrains. Subjects hailing from hilly areas of Uttarakhand were at 5-times increased risk of having anti-Toxoplasma antibodies [odds ratio(OR):5.0; 95% Confidence Interval(CI):2.31-10.82; p-value: <0.001] while subjects from plains had more than 3-times higher odds [OR:3.37; 95%CI: 1.58-7.21; p-value: 0.002]. Cases from western UP also showed more than 2-times higher odds of seropositivity at the univariate analysis[OR:2.25; 95%CI:1.04-4.88; p-value:0.041]; but did not reach statistical significance on the multivariate analysis (p-value: 0.282). Likewise, the chance of having T. gondii seropositivity was higher in people living in rural areas (39.8% vs. 31.1%) as compared to urban-dwelling people [OR:1.47; 95%CI: 0.97-2.23]. This statistical significance was retained in the multivariate model [OR:3.23; 95%CI:1.67-6.23; p-value: <0.001].

We also found a striking increase in the risk of toxoplasmosis in patients with co-morbidities. When compared to individuals without co-morbidities, those with other diseases had about 6 times higher odds of harboring anti-Toxoplasma antibodies [OR:6.19; 95%CI: 3.66—10.47].

There was no significant association between gender and Toxoplasma seropositivity. Hindus were 14% at higher risk of having seropositivity as compared to Muslims but it was not statistically significant (P-value: 0.640).

Consuming vegetarian diet was found to have a protective effect against Toxoplasma seropositivity as compared to non-vegetarian diets [OR:0.51; 95%CI:0.34-0.78; p-value: 0.002].

After adjustment of the relevant co-variates in the multivariable model (Table-2) age group of 40-50 years and >50 years, individuals residing in Uttarakhand (both plains and hilly terrain), living in rural areas and contiguity of co-morbidity showed independent association with thechance of Toxoplasma seropositivity. Pediatric age group (1-18 years) and vegetarian diet were found to have a lesser odds of T. gondii seropositivity after adjusting for other variables in the multivariate model.

## Discussion

To the best of our knowledge, this is a unique study from Uttarakhand to evaluate the seropositivity of anti-Toxoplasma antibodies in different groups from our state and adjoining areas.

### Toxoplasma seroprevalence

The rate of toxoplasma seropositivity in our state was higher as compared to other parts of India. The overall seropositivity rate in Uttarakhand was 36.88%, especially the hilly region recorded higher seropositivity (50%) followed by plains (40.3%). On the contrary, Dhumne et al. found a lower seroprevalence in northern India (UP=19.3%); however, data from Uttarakhand especially from hilly terrains is not available in the nationwide survey in India[4]. Another study reporteda seroprevalence of 21% from north India between 2004-2014 [6]. However, a higher prevalence rate of 45% was observed among pregnant women from North India by Singh et al.[13]. The high rate of seroprevalence as observed in our study clearly indicates the need for specific public health policies to prevent and control T. gondii infection in our state.

### Toxoplasma Seroprevalence in Uttarakhand

In logistic regression analysis too (both at univariable and multivariable level) people from Uttarakhand (both plains and hilly terrain) showed significantly higher rate of Toxoplasma seropositivity as compared to adjoining states. Also, the rural population was at a higher risk of acquiring toxoplasmosis (OR=3.23). Uttarakhand with an area of 53,483 sq. km is located at the foothills of the Himalayan mountain ranges, and topographically comprises mostly hills and mountains (46,035 sq.km) sharing boundary with Nepal and Tibet[14]. It has 6 National Park and 7 Wildlife Sanctuary and 4 Conservation Reserve, the most prominent beingCorbett National Park, Rajaji National Park, Nanda Devi National Park, etc. Total Forest cover area in the state constitutes 71% of the State’s geographical area which is habituated by 15 species of family Felidae and 5 species of family Canidae; besides about 3748 other species of mammals, birds, and reptiles [15]. Moreover, livestock and forest linkage are a major source of livelihood of people from hilly and rural areas. Thus, cohabitation with numerous species of animals might be an important factor for this significantly high T. gondii seroprevalence. Consistent with our findings, data from neighbouring Nepal also showed a higher seroprevalence of toxoplasmosis [16,17]. Rai et al. observed an overall seroprevalence of 65.3% in Nepal [16], while Sahimin et al. reported a high seroprevalence (by IgG) of 77.8% among migrant workers from Nepal in Malaysia [17].

### Association with age

Observation of significant increase in seropositivity with increasing age from pediatric to older age irrespective of gender indicated continued exposure to T. gondii throughout life. This finding was in conformity with various other studies [5,6,17-20]. In this study, significantly higher seroprevalence in the individuals above 40 years indicates a strong associationof infection among elderly. Of note the frequent seroconversion in elderly individuals is particularly a matter of concern due to frequent comorbidities and immunosuppression with age.

### Association with dietary habits

Although T. gondii may infect humans through intake of both vegetarian and non-vegetarian foods in the forms of ingested oocyst and tissue-cyst, we observed a significantly lesser seroprevalence in individuals with vegetarian diet. Similar observation was also reported by some earlier studies [16,18,20,21]. Method of cooking vegetables and lesser consumption of raw vegetables and salads by the natives might have a role in this protective effect. Nevertheless, 27.6% of vegetarians were also seropositive. Moreover, consumption of dried or undercooked goat or game meat dishes like ‘Suksa’ or ‘Bhunni’ by hilly and rural population might be a cause for higher seroprevalence among non-vegetarians.

### IgG and IgM seropositivity

More than one-third of the population (34.84%) showed IgG seropositivity which indicated previous or continued exposure of T. gondii. In addition, anti-toxoplasma IgG also showed a similar rising trend with increasing age (1year to >50 years). However, anti-toxoplasma IgM seropositivity was lower (6.33%) but remains a matter of concern for females of child-bearing age due to the possibility of congenital toxoplasmosis. Marginal male preponderance was observed but it did not reach statistical significance in our study. Although many studies reported higher seroprevalence in females, particularly in the child-bearing age [5,16,20,22], but contrarily some studies reported higher seroprevalence among males [18,19,23].

### Association with comorbidities

We observed a significant association between toxoplasma seropositivity and comorbid health conditions. However, due to the temporality of cross-sectional studies, significant statistical associations may not necessarily mean causality. The high strength of association may be due to the susceptibility of a particular group (e.g. diabetics, cancers, SLE) to various infectious diseases. Existence of some association could be expected based on present knowledge of certain diseases (e.g. congenital anomalies). Nonetheless, possible role of toxoplasmosis as a triggering factor responsible for the development of several clinical entities cannot be overruled as also suggested by other researchers [3,22].

Thus, we found that toxoplasmosis affects a major proportion of the population and yet it is often neglected in public health control programs targeting food-borne diseases [24]. Pre-and neo-natal screening and treatment, health education of targeted population (pregnant women, people from region of high endemicity), vaccination of farm animals, cats and other felids, decontamination of meat products are some of the effective interventions to reduce toxoplasmosis [25]. Countries like France and Austria have been successful in reducing the Toxoplasma gondii disease burden in humans with the implementation of gestational screening, educational programs, frequent re-testing and rapid treatment [26,27].

## Limitation of the study

The limitation of the present study was that being a retrospective hospital-based study many other putative risk factors like closeness to domestic or wild animals or familial clustering due to household effect etc. could not be analyzed due to unavailability of information. Therefore, larger community-based prospective studies can meet these gaps.

## Conclusion

In conclusion, seroprevalence of T. gondii antibodies were relatively higher in the Uttarakhand region, particularly in the rural and hilly terrain, indicating a necessity for the implementation of integrated public health strategies to prevent and control toxoplasmosis in this region. Increasing age, presence of comorbidities and non-vegetarian diet continue to be high risk factors for toxoplasmosis. Furthermore, larger community-based prospective studies are needed to fill the gaps of information identified in the present study.

### Future scope of the study

High prevalence of toxoplasmosis with possible risk factor’s like rural habitation, presence of comorbidity and dietary habit can be sufficient baseline data for a future community based prospective study in our population. This is important in the sense that there is no national screening/control program for toxoplasmosis which has the potential to be an important cause of enhanced mortality and morbidity in our population of Uttarakhand state. This is more so with increasing cases of immunodeficiency (e.g. HIV etc.)

### What is already known on this topic?

Multiple studies carried out earlier in variety of settings, concluded that the prevalence of toxoplasmosis is high (30-50%) in community. A list of risk factors for acquisition of infection was identified in various contexts (pregnancy, immunodeficiency etc.)

### What this study adds

This study reports the serological prevalence of anti-Toxoplasma antibodies in the hilly state of Uttarakhand and adjoining areas. A positive correlation of Toxoplasma seropositivty with increasing age, rural habitat of Uttarakhand and comorbidities was obtained. Such work in our population was not performed previously (published) and we hope, this will pave the way for a bigger prospective study in future which may indicate need for a screening/control program.

## Figures and Tables

**Table 1:** Seroprevalence of IgG and IgM antibodies to Toxoplasma gondii among 442 patients using ELISA

Antibodies	No. of seropositive, n	Seropositive (%)	95% CI (%) ^+^
**IgG+**	154	34.84	30.40 – 39.28
**IgM+**	28	6.33	4.06 – 8.61
**Both IgG+ IgM+**	19	4.30	2.41 – 6.19
Total seropositive *	**163**	**36.88**	**32.38 – 41.38**

CI: confidence interval, ELISA: Enzyme-Linked Immuno-Sorbent Assay

* positive for either of IgG or IgM

+ Confidence interval of ‘proportion’ is calculated using the normal approximation to the binomial calculation

**Table 2:** Stratified seroprevalence of anti-T. gondii antibodies and results of binomial logistic regression analysis of potential risk factors for seropositivity

Characteristics (n=442)	n (positive)	Prevalence in % [95% CI]	Univariate analysis		Multivariate analysis	
			OR [95% CI]	p-Value	aOR [95%CI]	p-Value
**Age (years)**			1.02 [1.0-1.03]	**0.012**	0.95 [0.87-1.03]	0.187
**Age groups**						
**Infants, <1 year (n=143)**	54	37.8 [29.8-45.7]	Ref		Ref	
**Pediatric age 1-18 years (n=61)**	12	19.7 [9.7-29.7]	0.40 [0.20-0.83]	**0.013**	0.04 [0.0-0.56]	**0.029**
**18-30 years (n=124)**	44	35.5 [27.1-43.9]	0.91 [0.55-1.49]	0.700	0.96 [0.24-2.25]	0.680
**30-40 years (n=68)**	25	36.8 [25.3-48.2]	0.96 [0.53-1.74]	0.889	1.34[0.43-2.63]	0.298
**40-50 years (n=24)**	14	58.3 [38.6-78.1]	2.31 [0.96-5.56]	**0.062**	2.08 [1.01-3.10]	**0.059**
**>50 years (n=22)**	14	63.6 [43.5-83.7]	2.88 [1.14-7.33]	**0.026**	2.98 [1.02-4.06]	**0.030**
**Place of Residence**						
**Adjacent states (n=60)**	10	16.7 [7.2-26.1]	Ref		Ref	
Uttarakhand plains (n=139)*	56	40.3 [32.1-48.4]	3.37 [1.58-7.21]	**0.002**	5.14 [2.20-12.02]	**0.000**
**Uttarakhand hills (n=114) †**	57	50.0 [40.8-59.2]	5.0 [2.31-10.82]	**0.000**	5.61 [2.14-14.68]	**0.000**
**West UP (n=129) ††**	40	31.0 [23.0-38.9]	2.25 [1.04-4.88]	**0.041**	1.61 [0.68-3.79]	0.282
**Sex**						
**Male (n=220)**	88	40 [33.5-46.5]	Ref		-	-
**Female (n=222)**	75	33.8 [27.6-40.0]	0.77 [0.52-1.13]	0.176	-	-
**Religion**						
**Muslim (n=64)**	22	34.4 [22.7-46.0]	Ref		-	**-**
**Hindu (n=366)**	137	37.4 [32.5-42.4]	1.14 [0.65-1.99]	0.640	-	**-**
**Others (n=12)**	4	33.3 [6.7-60.0]	0.96 [0.26-3.53]	0.944	-	**-**
**Rural versus Urban**						
**Urban (n=148)**	46	31.1 [23.6-38.5]	Ref		Ref	
**Rural (n=294)**	117	39.8 [34.2-45.4]	1.47 [0.97-2.23]	**0.074**	3.23 [1.67-6.23]	**0.000**
**Food habits**						
**Non-Vegetarian (n=272)**	116	42.6 [36.8-48.5]	Ref			
**Vegetarian (n=170)**	47	27.6 [20.9-34.4]	0.51 [0.34-0.78]	**0.002**	0.46 [0.28-0.75]	**0.002**
**Co-morbidities ^#^**						
**Absent (n=358)**	103	28.8 [24.1-33.7]	Ref			
**Present (n=84)**	60	71.4 [61.8-81.1]	6.19 [3.66-10.47]	**0.000**	8.64 [4.62-16.18]	**0.000**

Abbreviations: CI= Confidence Interval; OR= Odds ratio; Adj OR= Adjusted odds ratio; pos= number of positive cases

* Includes Rishikesh, Haridwar, Roorkee, Jwalapur, parts of Dehradun and Ranipokhari, parts of Kotdwar, Raiwala

† Includes Tehri-Garhwal, Pauri-Garhwal, Kumaun-ranges, Uttarkashi, Narendra Nagar, Rudraprayag, Mussoorie, Srinagar

††Includes Bijnor, Nazibabad, Muzzafarnagar, Shaharanpur, Kashipur

^#^Diabetes, HIV infection, past history of tuberculosis, SLE, viral hepatitis, congenital heart defects, other congenital anomalies, neoplasia and thalassemia.

## References

[1] Saadatnia G Golkar M. A review on human toxoplasmosis. Scand J Infect Dis. 2012;44:805-14. 10.3109/00365548.2012.693197 PMid: 22831461

[2] Robert-Gangneux F Dardé ML. Epidemiology of and diagnostic strategies for toxoplasmosis [published correction appears in Clin Microbiol Rev. 2012 Jul;25(3):583]. Clin Microbiol Rev. 2012;25(2):264-296. 10.1128/CMR.05013-11 PMid: PMCid: 22491772 PMC3346298

[3] Flegr J Prandota J Sovičková M Israili ZH. Toxoplasmosis-a global threat. Correlation of latent toxoplasmosis with specific disease burden in a set of 88 countries. PLoS One. 2014;9(3):e90203. 10.1371/journal.pone.0090203 PMid: PMCid: 24662942 PMC3963851

[4] Dhumne M Sengupta C Kadival G Rathinaswamy A Velumani A. National seroprevalence of Toxoplasma gondii in India. J Parasitol. 2007;93(6):1520-1521. 10.1645/GE-1159.1 PMid: 18314703

[5] Singh S Munawwar A Rao S Mehta S Hazarika NK. Serologic prevalence of Toxoplasma gondii in Indian women of child bearing age and effects of social and environmental factors. PLoSNegl Trop Dis. 2014;8(3):e2737 10.1371/journal.pntd.0002737 PMid: PMCid: 24675656 PMC3967963

[6] Mewara A Singh S Khurana S Gupta P Sehgal R. Seroprevalence of Toxoplasmosis at a Tertiary Care Centre in North India from 2004 to 2014. Indian J Med Microbiol. 2019 Jul-Sep;37(3):351-357. 10.4103/ijmm.IJMM_19_327 PMid: 32003332

[7] Sundar P Mahadevan A Jayshree RS Subbakrishna DK Shankar SK. Toxoplasma seroprevalence in healthy voluntary blood donors from Urban Karnataka. Indian J Med Res. 2007;126:50-5. PMid: . 17890824

[8] Luft BJ Remington JS. Toxoplasmic encephalitis in AIDS. Clin Infect Dis 1992;15:211-22. 10.1093/clinids/15.2.211 PMid: 1520757

[9] Mboera LEG Kishamawe C Kimario E Rumisha SF. Mortality Patterns of Toxoplasmosis and Its Comorbidities in Tanzania: A 10-Year Retrospective Hospital-Based Survey. Front Public Health. 2019;7:25.. 10.3389/fpubh.2019.00025 PMid: PMCid: 30838195 PMC6389597

[10] Jones JL Dubey JP. Foodborne toxoplasmosis. Clin Infect Dis. 2012;55(6):845-851. 10.1093/cid/cis508 PMid: 22618566

[11] Sonneville R Magalhaes E Meyfroidt G. Central nervous system infections in immunocompromised patients. Current Opinion in Critical Care. 2017 4;23(2):128-133. 10.1097/MCC.0000000000000397 PMid: 28169858

[12] Khan K Khan W. Congenital toxoplasmosis: an overview of the neurological and ocular manifestations. Parasitology International. 2018;67, 715-721. 10.1016/j.parint.2018.07.004 PMid: 30041005

[13] Singh S Pandit AJ. Incidence and prevalence of toxoplasmosis in Indian pregnant women: a prospective study. Am J Reprod Immunol. 2004 10;52(4):276-83. 10.1111/j.1600-0897.2004.00222.x PMid: 15494049

[14] Uttarakhand Biodiversity Board, Govt. of Uttarakhand. About Uttarakhand: about Uttarakhand. [online 2020] [cited 2021 March 28] Available from: URL: https://sbb.uk.gov.in/pages/display/93-about-uttarakhand

[15] Editor-Director,Zoological Survey of India, Kolkata, 2010. Fauna of Uttarakhand, State Fauna Series, 18(Part-l) 1-621. (Published by the Director, Zool. Surv. India, Kolkata). [online 2010] [cited 2021 March 28] Available from: URL: http://faunaofindia.nic.in/PDFVolumes/sfs/062/index.pdf

[16] Rai SK Matsumura T Ono K. High Toxoplasma seroprevalence associated with meat eating habits of locals in Nepal. Asia Pac J Public Health. 1999;11(2):89-93. 10.1177/101053959901100207 PMid: 11195164

[17] Sahimin N Lim YAL Ariffin F. Socio-demographic determinants of Toxoplasma gondii seroprevalence in migrant workers of Peninsular Malaysia. Parasit Vectors. 2017;10(1):238. 10.1186/s13071-017-2167-8 PMid: PMCid: 28506241 PMC5433061

[18] Wilking H Thamm M Stark K Aebischer T Seeber F. Prevalence, incidence estimations, and risk factors of Toxoplasma gondii infection in Germany: a representative, cross-sectional, serological study. Sci Rep. 2016;6:22551. 10.1038/srep22551 PMid: PMCid: 26936108 PMC4776094

[19] Fromont EG Riche B Rabilloud M. Toxoplasma seroprevalence in a rural population in France: detection of a household effect. BMC Infect Dis. 2009;9:76. 10.1186/1471-2334-9-76 PMid: PMCid: 19476609 PMC2696459

[20] Sakikawa M Noda S Hanaoka M. Anti-Toxoplasma antibody prevalence, primary infection rate, and risk factors in a study of toxoplasmosis in 4,466 pregnant women in Japan. Clin Vaccine Immunol. 2012;19(3):365-367. 10.1128/CVI.05486-11 PMid: PMCid: 22205659 PMC3294603

[21] Proctor EM Banerjee SN. The seroepidemiology of toxoplasmosis in the lower Fraser Valley of British Columbia. Can J Infect Dis. 1994;5(5):218-223. 10.1155/1994/586810 PMid: PMCid: 22346504 PMC3250829

[22] Lykins J Wang K Wheeler K Clouser F Dixon A Bissati KE. Understanding Toxoplasmosis in the United States Through “Large Data” Analyses. Clin Infect Dis. 2016;63(4):468-475. 10.1093/cid/ciw356 PMid: PMCid: 27353665 PMC4967610

[23] Jones JL Kruszon-Moran D Elder S Rivera HN. Toxoplasma gondii Infection in the United States, 2011-2014 [published correction appears in Am J Trop Med Hyg. 2018 Jul;99(1):241-242]. Am J Trop Med Hyg. 2018;98(2):551-557. 10.4269/ajtmh.17-0677 PMid: PMCid: 29260660 PMC5929212

[24] Jones JL Parise ME Fiore AE. Neglected parasitic infections in the United States: toxoplasmosis. Am J Trop Med Hyg. 2014;90(5):794-799. 10.4269/ajtmh.13-0722 PMid: PMCid: 24808246 PMC4015566

[25] Opsteegh M Kortbeek TM Havelaar AH van der Giessen JW. Intervention strategies to reduce human Toxoplasma gondii disease burden. Clin Infect Dis. 2015 1 1;60(1):101-7. 10.1093/cid/ciu721 PMid: 25225234

[26] Guigue N Léon L Hamane S. Continuous Decline of Toxoplasma gondii Seroprevalence in Hospital: A 1997-2014 Longitudinal Study in Paris, France [published correction appears in Front Microbiol. 2018 Nov 27;9:2814]. Front Microbiol. 2018;9:2369. 10.3389/fmicb.2018.02369 PMid: PMCid: 30344515 PMC6182058

[27] Bénard A Petersen E Salamon R Chêne G Gilbert R Salmi LR. European Toxo Prevention Study Group (EUROTOXO). Survey of European programmes for the epidemiological surveillance of congenital toxoplasmosis. Euro Surveill. 2008;13(15):. 10.2807/ese.13.15.18834-en PMid: PMCid: 18445459 PMC2740836

